# Luminescent Properties of Phosphonate Ester-Supported Neodymium(III) Nitrate and Chloride Complexes

**DOI:** 10.3390/molecules28010048

**Published:** 2022-12-21

**Authors:** Miriam Gerstel, Ingo Koehne, Johann Peter Reithmaier, Rudolf Pietschnig, Mohamed Benyoucef

**Affiliations:** 1 Institute of Nanostructure Technologies and Analytics (INA) and CINSaT, University of Kassel, Heinrich-Plett-Str. 40, 34132 Kassel, Germany; 2 Institute of Chemistry and Center for Interdisciplinary Nanostructure Science and Technology (CINSaT), University of Kassel, Heinrich-Plett-Str. 40, 34132 Kassel, Germany

**Keywords:** photoluminescence spectroscopy, lanthanide, phosphonate ester ligands, neodymium, X-ray

## Abstract

This study examines the synthesis of two geminal bisphosphonate ester-supported Ln^3+^ complexes [Ln(**L3**)_2_(NO_3_)_3_] (Ln = Nd^3+^ (**5**), La^3+^ (**6**)) and optical properties of the neodymium(III) complex. These results are compared to known mono-phosphonate ester-based Nd^3+^ complexes [Nd(**L1**/**L2**)_3_X_3_]_n_ (X = NO_3_^−^, n = 1; Cl^−^, n = 2) (**1**–**4**). The optical properties of Nd^3+^ compounds are determined by micro-photoluminescence (µ-PL) spectroscopy which reveals three characteristic metal-centered emission bands in the NIR region related to transitions from ^4^F_3/2_ excited state. Additionally, two emission bands from ^4^F_5/2_, ^2^H_9/2_ → ^4^I_J_ (J = 11/2, 13/2) transitions were observed. PL spectroscopy of equimolar complex solutions in dry dichloromethane (DCM) revealed remarkably higher emission intensity of the mono-phosphonate ester-based complexes in comparison to their bisphosphonate ester congener. The temperature-dependent PL measurements enable assignment of the emission lines of the ^4^F_3/2_ → ^4^I_9/2_ transition. Furthermore, low-temperature polarization-dependent measurements of the transitions from R_1_ and R_2_ Stark sublevel of ^4^F_3/2_ state to the ^4^I_9/2_ state for crystals of [Nd(**L3**)_2_(NO_3_)_3_] (**5**) are discussed.

## 1. Introduction

Luminescent lanthanide(III) (Ln^3+^) ions have attracted considerable research interest due to their versatile photophysical properties [1,2,3,4,5], which are related to their 4f electrons: Due to their small radial distribution, 4f electrons exhibit minimal interaction and little involvement in chemical bonding with surrounding ligands, since they are effectively shielded by electrons of the 5s and 5p shell. Thus, Ln^3+^ luminescence exhibits characteristic narrow emission bands (FWHM < 10 nm) [6,7] along with relatively long emission lifetimes in a micro- or millisecond range [8,9,10]. This UV/Vis-to-NIR photoluminescence results from intra-configurational 4f-4f transitions [11] which are Laporte forbidden, but partially permitted by mixing of 4f and 5d orbitals or with charge transfer states of neighboring ligands [6,12].

Luminescence of Ln^3+^ ions has received considerable attention due to potential applications in a variety of technological fields such as light-emitting diodes [13,14,15], bioimaging [16,17,18,19,20], optical telecommunication [21,22,23], and luminescent ratiometric thermometers [24,25,26,27,28,29,30]. Especially Nd^3+^ ions have attracted interest for the development of diode-pumped solid-state lasers based on Nd-doped crystals [31,32,33,34,35,36], used for medical applications [37,38] and material processing [39,40,41]. We previously reported the synthesis of mono-phosphonate ester-supported Ln^3+^ (Ln=La, Nd, Dy, Er) complexes including their structural and optical characterizations [42]. Starting with various mono-phosphonate esters featuring varied aromatic residues, mono- or dimeric lanthanide complexes of the type [Ln(**L**)_3_X_3_]_n_ (**1**–**4**) (L = phosphonate ester ligand; X = NO_3_^−^, n = 1; Cl^−^, n = 2) were synthesized. Room temperature (RT) absorption as well as luminescence spectra of Dy^3+^ and Nd^3+^ complexes were reported. In this work, we present additional synthesis and characterization of two geminal bisphosphonate ester-supported Ln^3+^ complexes [Ln(**L3**)_2_(NO_3_)_3_] (Ln = Nd^3+^ (**5**), La^3+^ (**6**)) (for preparation of **L3** see [43]) as well as a comparison of the photoluminescence properties of the Nd^3+^ derivative **5** to compounds **1**–**4**. Micro-photoluminescence (µ-PL) studies of the complexes at RT reveal the characteristic emission bands of neodymium(III) ions which are centered around 890 nm, 1060 nm, and 1350 nm, corresponding to the ^4^F_3/2_ → ^4^I_9/2_, ^4^F_3/2_ → ^4^I_11/2_, and ^4^F_3/2_ → ^4^I_13/2_ transitions, respectively. Furthermore, two transition bands assigned to transitions from ^4^F_5/2_ and ^2^H_9/2_ excited states to ^4^I_J_ (J = 11/2, 13/2) were detected. Evaluation of RT measurements of equimolar complex solutions provide insight into the emission strength of the various complexes. High-resolution µ-PL measurements of solid bulk material at liquid helium temperature (5 K) give detailed information on the three characteristic emission bands of neodymium(III) ions corresponding to transitions from the ^4^F_3/2_ manifold. Low-temperature polarization-dependent µ-PL measurements reveal information about the crystal orientation of the complex [Nd(**L3**)_2_(NO_3_)_3_] (**5**).

## 2. Results

### 2.1. Mono-Phosphonate Ester-Based Nd^3+^ Complexes **1**–**4** and the Geminal Bisphosphonate Ester-Supported Complexes **5** (Nd^3+^) and 6 (La^3+^)

**Scheme 1:**
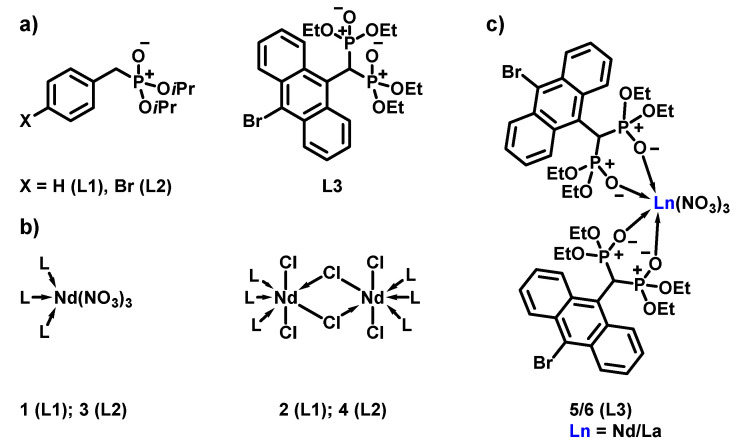
(**a**) Overview on the mono-phosphonate ester ligands **L1** and **L2** and the anthracene-based geminal bisphosphonate ester **L3**. (**b**) Schematic structures of **L1**- and **L2**-supported complexes **1**–**4**: Monomeric Nd(NO_3_)_3_-based complexes **1** and **3** and dimeric NdCl_3_-based species **2** and **4**. (**c**) Monomeric structure of **L3**-supported Ln(NO_3_)_3_ (Ln = Nd^3+^, La^3+^) compounds **5** and **6**.

**Figure 1:**
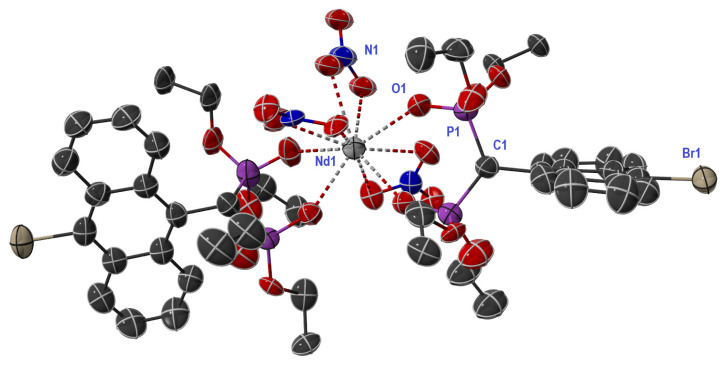
The molecular structure of complex [Nd(**L3**)_2_(NO_3_)_3_] (**5**) which is isostructural to the solid-state structure of lanthanum(III) derivative [La(**L3**)_2_(NO_3_)_3_] (**6**). Anisotropic displacement parameters are depicted at the 50% probability level. Hydrogen atoms are omitted for clarity. Symmetry transformations used to generate equivalent atoms: #1: −x + 1, y, −z + 3/2. Selected bond lengths [Å] and angles [°] of **5** and **6** (If there is more than one value for a considered bond length or angle, merged values are given): Ln–O_P=O_ (**5**: 2.443(5); **6**: 2.525(5)), Ln–O_NO3_ (**5**: 2.570(11); **6**: 2.629(18)), P=O (**5**: 1.466(5); **6**: 1.467(5)), P–C (**5**: 1.779(7); **6**: 1.788(7)), P–C–P (**5**: 118.4(4); **6**: 117.8(4)), O_P=O_–Ln–O_P=O_ (**5**: 72.5(16); **6**: 71.4(16)).

The ligand platforms **L1**–**L3** as well as complexes **1**–**4** are synthesized according to literature procedures (Scheme 1a,b) [15,16]. For the preparation of the latter, three equivalents of ligand **L1** or **L2** are combined with a [Nd(H_2_O)_6_X_3_] (X = NO_3_^−^ or Cl^−^) precursor in EtOH solution. Compounds [Ln(**L3**)_2_(NO_3_)_3_] (Ln = Nd^3+^ (**5**) or La^3+^ (**6**)) are prepared in the same manner, but under the addition of only two equivalents of geminal bisphosphonate ester **L3** and are obtained as pale-yellow to yellow solids. The lanthanum(III) derivative is prepared in order to have additional access to NMR spectroscopy as a powerful analytic tool. In comparison to free **L3** which exhibits a ^31^P{^1^H} NMR resonance at 19.4 ppm [16], complex **6** shows a slightly high field shifted signal at 18.8 ppm. The same observation can be made in the ^1^H NMR spectrum for the corresponding methine bridge proton of the P–C–P moiety. The triplet resonance at 5.48 ppm (^2^*J*_PH_ = 30.8 Hz) of free **L3** is shifted to 5.39 ppm in complex **6**. The P=O vibration at 1252 cm^−1^ of free **L3** becomes red-shifted to 1222 cm^−1^ (**5**) and 1221 cm^−1^ (**6**) upon coordination to an electron deficient metal ion corresponding to a slight weakening of the P=O bond (SI, Figures S5 and S6). In general, the P=O vibrations are in good agreement with those observed for other related phosphonate ester-supported lanthanide complexes [42,44,45,46,47]. Crystals of [Nd(**L3**)_2_(NO_3_)_3_] (**5**) and [La(**L3**)_2_(NO_3_)_3_] (**6**) are obtained from vapor diffusion of pentanes into saturated tetrahydrofuran (THF) solutions of each complex. The compounds are isostructural, both crystallizing in the monoclinic space group *C*2/*c* as well as showing half a molecule in the asymmetric unit. The molecular structure of [Nd(**L3**)_2_(NO_3_)_3_] (**5**) is exemplarily shown in Figure 1. Complexes **5** and **6** adopt a doubly-capped square-antiprismatic geometry in a ten-fold all-O coordination.

The Ln–O_P=O_ (**5**: 2.443(5); **6**: 2.525(5)) and Ln–O_NO3_ (**5**: 2.570(11); **6**: 2.629(18)) distances increase going from complex [Nd(**L3**)_2_(NO_3_)_3_] (**5**) to [La(**L3**)_2_(NO_3_)_3_] (**6**) in accordance with an increasing ionic radius from Nd^3+^ (1.11 Å) to La^3+^ (1.16 Å) [48]. The free ligand **L3** shows merged P=O and P–C bond lengths of 1.462(4) Å and 1.806(6) Å, respectively. [16] In contrast, the P=O (**5**: 1.466(5) Å; **6**: 1.467(5) Å) distance becomes slightly elongated while the P–C bond (**5**: 1.779(7) Å; **6**: 1.788(7) Å) becomes somewhat shortened upon lanthanide ion coordination. To properly host the metal ions, the observed P–C–P angle (**5**: 118.4(4) °; **6**: 117.8(4)) is less acute than in the free ligand system **L3** (113.7(3)). The O_P=O_–Ln–O_P=O_ (**5**: 72.5(16); **6**: 71.4(16)) angle expectedly decreases when switching from the smaller Nd^3+^ to the bigger La^3+^ ion.

### 2.2. Photoluminescence Properties

#### 2.2.1. Room Temperature Emission Properties of Nd^3+^ Complexes **1**–**5** from Amorphous Solids and Solutions

**Figure 2:**
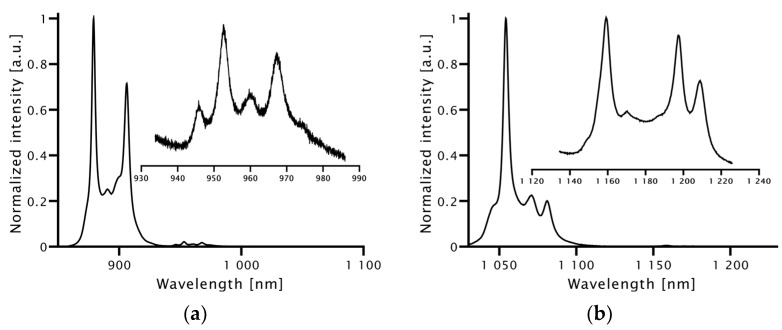
Normalized emission spectra of [Nd(**L1**)_3_Cl_3_]_2_ (**2**) obtained from amorphous bulk material at room temperature (RT): (**a**) ^4^F_3/2_ → ^4^I_9/2_ transition and ^4^F_5/2_, ^2^H_9/2_ → ^4^I_11/2_ (inset). (**b**) ^4^F_3/2_ → ^4^I_11/2_ transition and ^4^F_5/2_, ^2^H_9/2_ → ^4^I_13/2_ (inset).

Neodymium(III) complex [Nd(**L3**)_2_(NO_3_)_3_] (**5**) exhibits a broad absorption band in the UV range due to ligand absorption as well as sharp absorption bands between 500 to 850 nm, characteristic of Nd^3+^ ions (see Figure S7 in SI file). Excitation of the synthesized Nd^3+^ complexes at 750 nm, which is resonant with the ^4^I_9/2_ → ^4^F_7/2_, ^4^S_3/2_ transition, results in the detection of three emission bands in the NIR region (centered around 890 nm, 1060 nm, and 1350 nm). These are associated with the electronic transitions ^4^F_3/2_ → ^4^I_9/2_, ^4^F_3/2_ → ^4^I_11/2_, and ^4^F_3/2_ → ^4^I_13/2_, respectively. Pumping into the ^4^F_7/2_, ^4^S_3/2_ levels also enable the detection of two emission lines from the ^4^F_5/2_, ^2^H_9/2_ excited states (^4^F_5/2_, ^2^H_9/2_ → ^4^I_J_) centered around 960 nm (J = 11/2) and 1180 nm (J = 13/2). However, following non-radiative decay and due to the small energy gap between ^4^F_5/2_, ^2^H_9/2_ levels, and ^4^F_3/2_, emission from the lower excited state represents the dominant process. Figure 2 depicts RT emission spectra of [Nd(**L1**)_3_Cl_3_]_2_ (**2**) highlighting the relatively weak transition bands from the ^4^F_5/2_, ^2^H_9/2_ states as insets, which were detected also for the other Nd^3+^ complexes. These emission bands from ^4^F_5/2_, ^2^H_9/2_ states cannot be observed from conventional laser materials such as Nd:YAG, but were observed from Nd-doped lead halides [49,50,51]. Lead halides represent solid-state host materials with low maximum phonon energy and therefore less quenching of luminescence from ^4^F_5/2_, ^2^H_9/2_ excited states.

**Figure 3:**
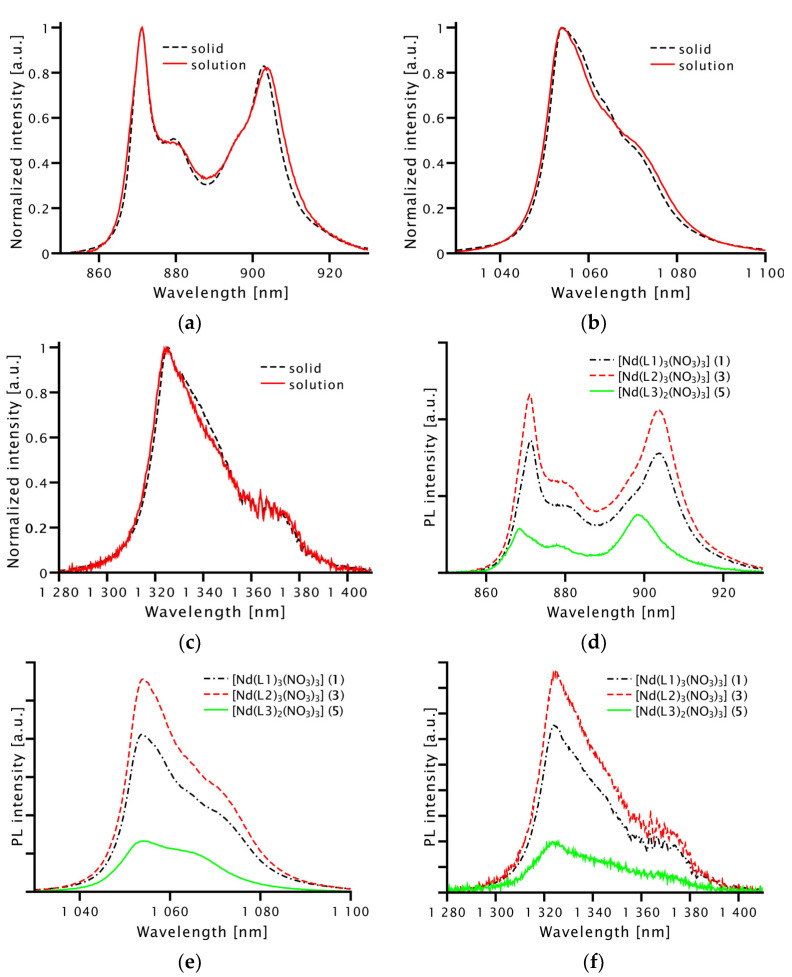
The normalized emission spectra of complex [Nd(**L2**)_3_(NO_3_)_3_] (**3**) obtained from amorphous bulk material (dashed line) and a solution in dry dichloromethane (DCM) (solid line, c = 4 × 10^−3^ mol/L) related to the transition from ^4^F_3/2_ to ^4^I_9/2_ (**a**),^4^I_11/2_ (**b**) and ^4^I_13/2_ (**c**). RT photoluminescence (PL) spectra of [Nd(**L1**)_3_(NO_3_)_3_] (**1**) (dot-dashed line), [Nd(**L2**)_3_(NO_3_)_3_] (**3**) (dashed line), and [Nd(**L3**)_2_(NO_3_)_3_] (**5**) (solid line) in dry DCM (c = 4 × 10^−3^ mol/L) of ^4^F_3/2_ → ^4^I_9/2_ (**d**), ^4^F_3/2_ → ^4^I_11/2_ (**e**), and ^4^F_3/2_ → ^4^I_13/2_ (**f**) transition. For data acquisition, laser power and excitation duration were kept constant. The spiky signal of the ^4^F_3/2_ → ^4^I_13/2_ transition (**c**,**f**) between 1350 nm and 1380 nm is related to setup noise.

Investigation of solutions of equimolar concentration enables comparison of emission intensities of the various complexes. For comparison, Figure 3a–c firstly represents the emission spectra obtained from amorphous bulk material [Nd(**L2**)_3_(NO_3_)_3_] (**3**) and of the complexes dissolved in dry dichloromethane (DCM). Only a minor shift in the emission bands was observed.

Since the mono-phosphonate ester-supported neodymium chloride complexes are less soluble in DCM, only nitrate-based complexes with mono-phosphonate and geminal bisphosphonate esters are considered. The complexes are dissolved in dry DCM (*c* = 4 × 10^−3^ mol/L) and their optical characteristics were investigated at RT using µ-PL spectroscopy (same laser power and acquisition time for all three complexes). Since there is less rotation and vibrational modes along the Nd-O bonds, geminal bisphosphonate ester-based complexes are more rigid than their mono-phosphonate ester congeners. As a result, enhanced luminescence intensity can be anticipated for complexes based on a geminal bisphosphonate ester such as **L3**. In contrast, the emission spectra shown in Figure 3d–f demonstrate that higher emission intensities are detected in the case of the mono-phosphonate esters-supported complexes, with [Nd(**L2**)_3_(NO_3_)_3_] (**3**) exhibiting the most intense emissions. The salient emission intensity of [Nd(**L2**)_3_(NO_3_)_3_] (**3**) is about four times higher compared to [Nd(**L3**)_2_(NO_3_)_3_] (**5**). This observation is possibly a consequence of better ligand-to-metal charge transfer (LMCT) for the evaluated mono-phosphonate ester ligands compared with the parent geminal bisphosphonate ligand system **L3**.

#### 2.2.2. Liquid Helium Temperature Emission Properties of Nd^3+^ Complexes **1**–**5**

**Figure 4:**
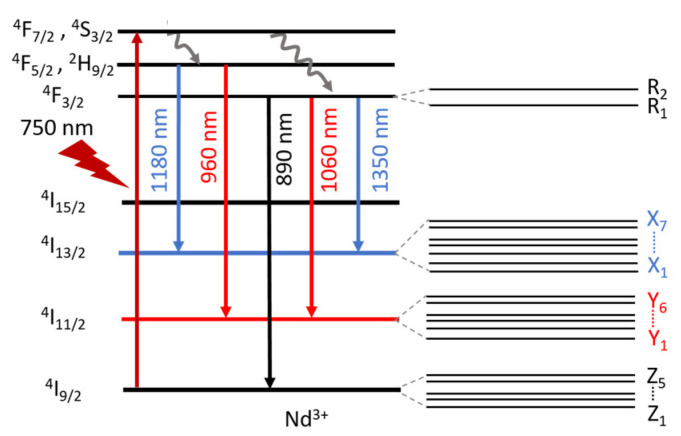
Nd^3+^ ion energy level diagram: The absorption transition ^4^I_9/2_ → ^4^F_7/2_, ^4^S_3/2_ (excitation at 750 nm) is followed by non-radiative decay to ^4^F_5/2_, ^2^H_9/2_ and ^4^F_3/2_ states, respectively. With depopulation of these states’ emission bands centered around 960 nm (^4^F_5/2_, ^2^H_9/2_ → ^4^I_11/2_) and 1180 nm (^4^F_5/2_, ^2^H_9/2_ → ^4^I_13/2_) as well as around 890 nm, 1060 nm, 1350 nm occur. Latter ones correspond to the ^4^F_3/2_ →^4^I_9/2_, ^4^F_3/2_ → ^4^I_11/2_, and ^4^F_3/2_ → ^4^I_13/2_ transitions, respectively. Due to the ligand field splitting, the ^4^F_3/2_, ^4^I_9/2_, ^4^I_11/2_, and ^4^I_13/2_ states are split into two (R_1_, R_2_), five (Z_1_–Z_5_), six (Y_1_–Y_6_) and seven (X_1_–X_7_) Stark sublevels, respectively.

At liquid helium temperature (5 K), narrow emission lines (FWHM = 1.1 nm–4.6 nm) can be observed as compared to RT measurements. Generally, there are 10, 12, and 14 transition lines resulting from transitions from the ^4^F_3/2_ manifold to the ^4^I_9/2_, ^4^I_11/2_, and ^4^I_13/2_ manifolds, respectively, under the assumption of a non-cubic symmetry around the Nd^3+^ ion, which has an odd number of electrons (Kramers ions [52,53]). This can be rationalized by using the energy level diagram displayed in Figure 4. It illustrates how the ^4^F_3/2_ state is divided into upper (R_2_) and lower (R_1_) Stark sublevels, whereas the ^4^I_9/2_, ^4^I_11/2_, and ^4^I_13/2_ states are split into five (Z_1_-Z_5_), six (Y_1_-Y_6_) and seven (X_1_-X_7_) sublevels, respectively.

**Figure 5:**
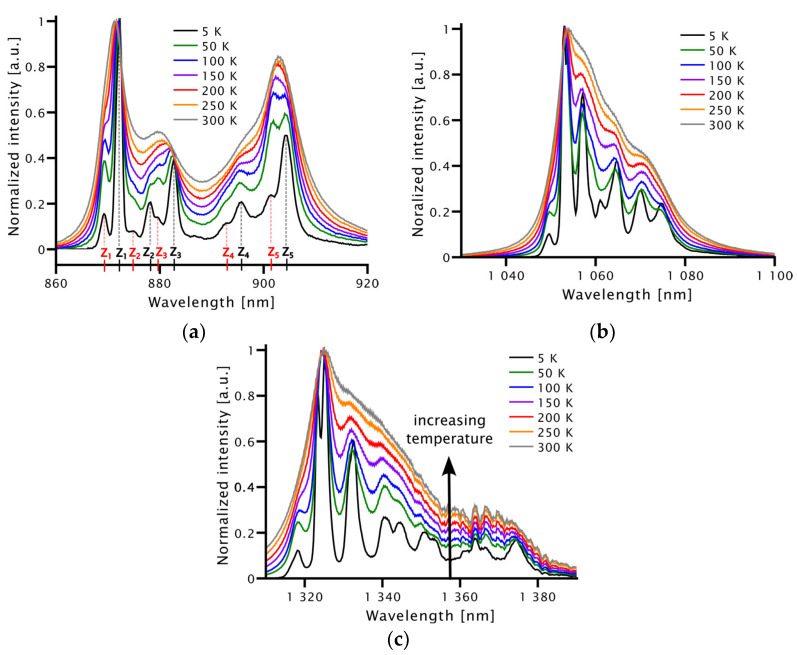
Temperature-dependent emission spectra of amorphous bulk material of [Nd(**L2**)_3_(NO_3_)_3_] (**3**) assigned to ^4^F_3/2_ → ^4^I_9/2_ (**a**), ^4^F_3/2_ → ^4^I_11/2_ (**b**), and ^4^F_3/2_ → ^4^I_13/2_ (**c**) transitions, respectively. For the ^4^F_3/2_ → ^4^I_9/2_ transition the emission lines are labeled: Transition lines from R_1_ are marked in black (Z_1_-Z_5_) and from R_2_ to Z_1_-Z_5_ in red. The spiky signal of the ^4^F_3/2_ → ^4^I_13/2_ transition in (**c**) between 1350 nm and 1380 nm is related to setup noise.

Figure 5 illustrates the temperature-dependent emission spectra of complex [Nd(**L2**)_3_(NO_3_)_3_] (**3**). At low temperature (5 K), ten emission lines of the ^4^F_3/2_ → ^4^I_9/2_ transition can be observed, which overlap strongly at higher temperatures due to the broadening of electron-phonon interactions. Despite low-temperature measurements, ^4^F_3/2_ → ^4^I_11/2_ and ^4^F_3/2_ → ^4^I_13/2_ transitions are less resolved. This is due to the spectral overlap between the emission lines since they lie close together.

**Figure 6:**
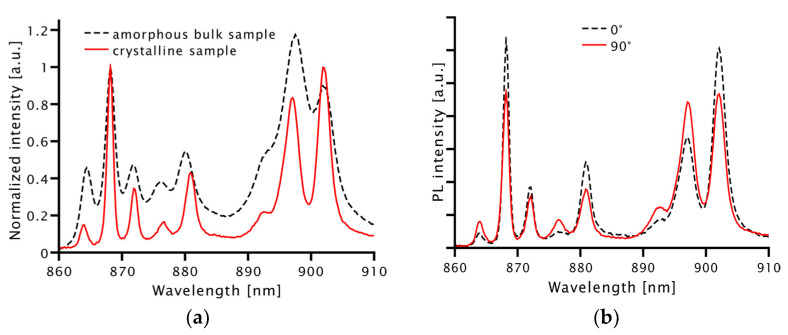
(**a**) The normalized PL emission spectra at 5 K of [Nd(**L3**)_2_(NO_3_)_3_] (**5**) corresponding to ^4^F_3/2_ → ^4^I_9/2_ transition from amorphous bulk sample (dashed line) and crystalline sample (solid line). (**b**) Low-temperature (5 K) PL spectra of crystalline [Nd(**L3**)_2_(NO_3_)_3_] (**5**) of the ^4^F_3/2_ → ^4^I_9/2_ transition taken at a polarization angle of 0° and 90°.

As the morphology has a significant impact on the emission spectrum, crystals of the geminal bisphosphonate ester-supported compound [Nd(**L3**)_2_(NO_3_)_3_] (**5**) are investigated in this respect. The normalized PL spectra of the amorphous bulk and crystalline complexes are depicted in Figure 6a. As expected, the amorphous bulk material has a wider linewidth than the crystalline sample due to structural disorder.

When a perfect crystal considered under ideal conditions is excited with a laser, the emission bands have a narrow Lorentzian shape since all emitting molecules have the same orientation in the crystal. Due to differences in the local environment of the Nd^3+^ centers in amorphous bulk samples and grown crystals, broadened Gaussian band shapes are observed [54]. As described by Lenz et al. [54] the PL intensity of transition lines is strongly affected by the crystal orientation. This orientation-dependence was also observed for crystalline [Nd(**L3**)_2_(NO_3_)_3_] (**5**) when applying a polarizer in front of the detector. As the polarization angle increases from 0° to 90°, the intensity of the transition lines associated with the R_1_ sublevel decrease, whereas transition lines associated with the R_2_ sublevel increase, as illustrated in Figure 6b.

## 3. Discussion

According to Figure 4, ten, twelve and fourteen emission lines are expected for the ^4^F_3/2_ → ^4^I_9/2_, ^4^F_3/2_ → ^4^I_11/2_, and ^4^F_3/2_ → ^4^I_13/2_ transitions, respectively. Due to spectral overlap, not all transition lines can be resolved in the emission spectra as demonstrated by the ^4^F_3/2_ → ^4^I_9/2_ transition of [Nd(**L3**)_2_(NO_3_)_3_] (**5**), where only eight of ten emission lines can be observed (Figure 6a). However, temperature-dependent PL measurements can be used to assign the emission lines. At RT, the R_2_ Stark sublevel is easily populated due to the small energy difference between R_1_ and R_2_ [55,56]. With decreasing temperature, R_2_ is less populated leading to decreasing PL intensity of the corresponding emission lines. Figure 5a illustrates the increase in emission intensities of transition lines centered at 869.3 nm, 875.3 nm, 879.8 nm, 892.6 nm, and 901.2 nm with rising temperature for the ^4^F_3/2_ → ^4^I_9/2_ transition of [Nd(**L2**)_3_(NO_3_)_3_] (**3**). A radiative depopulation of the R_2_ Stark sublevel of the ^4^F_3/2_ state to the ^4^I_9/2_ manifold results in the emission of these lines. Increased intensity of emission lines related to transitions from the R_1_ sublevel centered at 878.1 nm, 895.7 nm, and 904.6 nm may be caused by spectral overlap with R_2_ Stark sublevel emission lines. The emission bands of the ^4^F_3/2_ → ^4^I_11/2_, and ^4^F_3/2_ → ^4^I_13/2_ transitions were not assigned, as the emission lines overlap strongly even at low temperatures.

**Figure 7:**
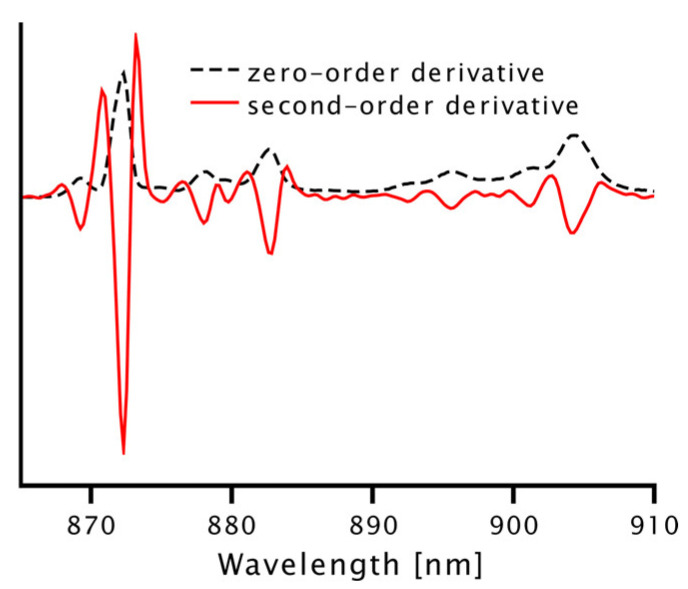
Zero-order (dashed line) and second-order derivative spectrum (solid line) of [Nd(**L2**)_3_(NO_3_)_3_] (**3**) corresponding to the ^4^F_3/2_ → ^4^I_9/2_ transition.

Due to spectral overlap and low intensity of some of the emission lines, its peak positions (*λ_max_*) cannot be determined precisely. A powerful tool for resolving overlapping spectral bands is the so-called derivative spectroscopy [57,58], which gives detailed information about emission lines and *λ_max_* values. Figure 7 depicts the zero-order (dashed line) PL spectrum of [Nd(**L2**)_3_(NO_3_)_3_] (**3**) (recorded at 5 K) and its second-order derivative (D2) spectrum (solid line). When compared to the original (zero-order) PL spectrum, the D2 spectrum’s peaks are reversed, revealing minima at *λ_max_* of the zero-order spectrum. In addition, a positive satellite band is also present on either side of each dip. In general, sharp peaks of zero-order spectra become even narrower in D2 spectra, while broad peaks will be flattened, leading to a reduction in broad background but also to unwanted enhancement of sharp noise-signals. Thus, PL spectra were smoothed to increase the signal-to-noise ratio. The *λ_max_* values of the Nd^3+^ complexes are derived from the second-order derivative spectra and are summarized in Table 1. Values for several transition lines are not reported in the table, because *λ_max_* of the transition lines in case of [Nd(**L2**)_3_CL3]_2_ (**4**) and [Nd(**L3**)_2_(NO_3_)_3_] (**5**) overlapped and could not be resolved properly. Only seven transition lines can be detected in case of [Nd(**L1**)_3_Cl_3_]_2_ (**2**), which is insufficient for a valid assignment.

**Figure 8:**
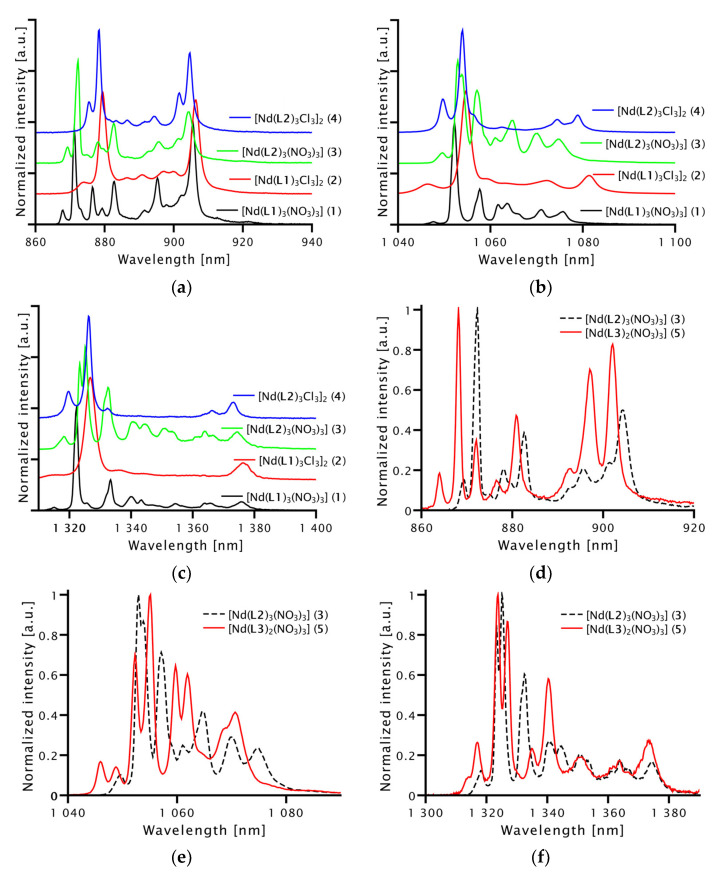
The normalized low-temperature (5 K) PL spectra of amorphous bulk [Nd(**L1**)_3_(NO_3_)_3_] (**1**) (black), [Nd(**L1**)_3_Cl_3_]_2_ (**2**) (red), [Nd(**L2**)_3_(NO_3_)_3_] (**3**) (green), and [Nd(**L2**)_3_Cl_3_]_2_ (**4**) (blue) for the ^4^F_3/2_ → ^4^I_9/2_ (**a**), ^4^F_3/2_ → ^4^I_11/2_ (**b**), and ^4^F_3/2_ → ^4^I_13/2_ (**c**) transitions, respectively. (**d**–**f**) Low-temperature (5 K) PL spectra of mono-phosphonate ester-supported compound [Nd(**L2**)_3_(NO_3_)_3_] (**3**) (dashed line) and geminal bisphosphonate ester complex [Nd(**L3**)_2_(NO_3_)_3_] (**5**) (solid line) for the three different emission bands.

As previously stated, the 4f shell of lanthanides is well shielded by electrons of the 5s and 5p orbitals resulting in only minor influence from neighboring ligands. However, for Stark level splitting, ligand parameters such as interatomic distances and electric charge are critical [54]. As a result, nitrate and chloride anions have a significant influence on the PL spectra. Figure 8a–c compares the 5 K emission bands associated with the three NIR transitions of Nd^3+^ complexes **1**–**4**. The emission bands of the monomeric NO_3_^−^ and dimeric Cl^−^ based Nd^3+^ complexes under investigation are similar, nevertheless, there are two noteworthy differences: First, NO_3_^−^ based complexes exhibit emission lines for the ^4^F_3/2_ → ^4^I_9/2_ transition that start at shorter wavelengths and are spread across a wider spectral range. Second, the transition lines of the neodymium(III) chloride complexes, apart from the two outer lines of each transition band, are much less prominent than those of the nitrate-based congeners. It appears that the different organic ligands do not significantly influence the µ-PL spectrum, as similar spectra were observed for [Nd(**L1**)_3_(NO_3_)_3_] (**1**) and [Nd(**L2**)_3_(NO_3_)_3_] (**3**), and [Nd(**L1**)_3_Cl_3_]_2_ (**2**) and [Nd(**L2**)_3_Cl_3_]_2_ (**4**), respectively.

Figure 8d–f compares the low-temperature (5 K) PL spectra of mono-phosphonate ester-supported complexes [Nd(**L2**)_3_(NO_3_)_3_] (**3**) (dashed line) and [Nd(**L3**)_2_(NO_3_)_3_] (**5**) (solid line). The geminal bisphosphonate ester complex **5** exhibits a redshift in emission compared to compounds with mono-phosphonate ester ligands, such as **3**. The R_2_ → Z_1_ transition line for **3** and **5** is shifted by 3.7 nm (Figure 8d). In both NO_3_ based complex types, the transition line shape is similar.

**Figure 9:**
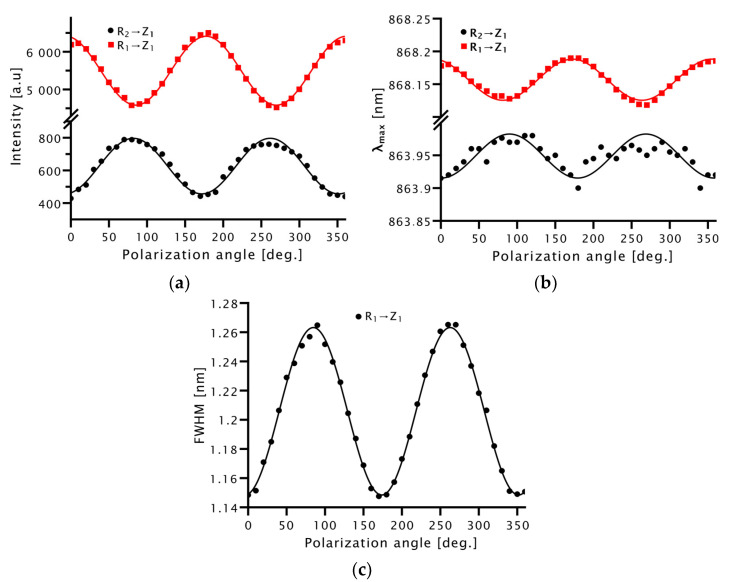
(**a**) Emission intensities of the R_2_ → Z_1_ and R_1_ → Z_1_ transition lines of compound [Nd(**L3**)_2_(NO_3_)_3_] (**5**); (**b**) λ_max_ of the R_2_ → Z_1_ and R_1_ → Z_1_ transition lines of **5**; (**c**) FWHM of the R_1_ → Z_1_ transition line of **5** as a function of the polarization angle.

In line with the already mentioned orientation-dependence of the PL intensity of transition lines for crystalline samples, we explored this aspect for the geminal bisphosphonate ester complex [Nd(**L3**)_2_(NO_3_)_3_] (**5**) by introducing a polarizer in front of the detector. Since the first two transition lines of ^4^F_3/2_ → ^4^I_9/2_ transition are spectrally most isolated, the following investigations focus on the R_2_ → Z_1_ and R_1_ → Z_1_ transition lines. The relative emission intensities as a function of the polarization angle, as extracted from careful fits of many spectra, are shown in Figure 9a. As can be seen, an increase in peak intensity corresponds to transitions from the upper Stark sublevel R_2_ while a decrease in peak intensity corresponds to the transition from the lower Stark R_1_ sublevel and *vice versa*. Figure 9b shows the transition energies of the two transition lines as a function of the linear polarization angle. The oscillatory behavior of both lines stems from two perpendicularly linearly polarized components. The two transitions show anticorrelated shifts when changing the polarization angle, confirming the above assigned transitions. While polarized emission has been observed, the exact correlation between polarization and crystal orientation has not yet been examined. It is necessary to conduct further investigations, which is beyond the scope of this study. The FWHM was also found to be polarization-dependent as shown in Figure 9c. The two transitions show as well anticorrelated broadening when changing the polarization angle. Here only the R_1_ → Z_1_ transition line is plotted. Since the peak intensity of the R_2_ → Z_1_ transition is weak, the fitting of the emission line was not unambiguous, therefore it is not shown.

## 4. Materials and Methods

Starting materials for synthesis were purchased commercially and were used as received, unless stated otherwise. The ligands **L1**–**L3** as well as complexes **1**–**4** have been prepared according to literature protocols [42,43]. NMR experiments were performed with a Varian 500 MHz spectrometer, and spectra were processed with MestReNova (v11.0.4-18998, Mestrelab Research S.L.). ^1^H- and ^13^C NMR spectra are referenced relative to TMS using the residual solvent signals as internal standards [59]. IR spectra were recorded with a diamond probe ATR IR spectrometer by Bruker. Elemental analyses were performed using a HEKAtech Euro EA-CHNS elemental analyzer. For analyses, samples were prepared in tin cups with V_2_O_5_ as an additive to ensure complete combustion.

### 4.1. General Procedure for the Preparation of Geminal Bisphosphonate Ester-Supported Ln^3+^ (Ln = Nd^3+^ (**5**), La^3+^ (**6**)) Complexes

[Ln(H_2_O)_6_(NO_3_)_3_] (Ln = Nd^3+^, La^3+^; 1.00 mmol, 1.00 eq.) is dissolved in a vial in EtOH (15 mL). Ligand **L3** (1.09 g, 2.00 mmol, 2.00 eq.) is dissolved in a round-bottom flask in EtOH (15 mL). The lanthanide precursor solution is added to the ligand solution under stirring and the mixture is stirred at RT overnight (~16 h). The formed precipitate is recovered via percolation over a pleated filter, the filter cake is washed with small amounts of −20 °C EtOH and subsequently, air dried. The complexes are isolated as pale-yellow solids in non-optimized yields of 58% (**5**) and 59% (**6**). *[Nd(**L3**)_2_(NO_3_)_3_] (**5**):* IR (ATR) $\tilde{\nu }$ = 1222 (P=O), 1100 (P–OEt) cm^−1^; Anal. Calcd for C_46_H_58_Br_2_N_3_NdO_21_P_4_: C, 38.99; H, 4.13; N, 2.97. Found: C, 38.99; H, 4.18; N, 2.93; *[La(**L3**)_2_(NO_3_)_3_] (**6**):* ^1^H-NMR (500 MHz, DMSO-d_6_): *δ* = 9.19 (d, 2H, ^3^*J*_HH_ = 9.0 Hz, H4), 8.57 (dd, 2H, ^3^*J*_HH_ = 6.4, ^4^*J*_HH_ = 3.6 Hz, H1), 8.49 (d, 2H, ^3^*J*_HH_ = 8.8 Hz, H8), 8.37 (dd, 2H, ^3^*J*_HH_ = 6.4, ^4^*J*_HH_ = 3.6 Hz, H5), 7.75–7.69 (m, 6H, H2 + H6, H3), 7.63–7.59 (m, 2H, H7) 5.39 (t, 2H, ^2^*J*_PH_ = 30.3 Hz, CH), 4.18–4.11 (m, 8H, C*H*_2_CH_3_), 3.86–3.74 (m, 2H, C*H*_2_CH_3_), 3.68–3.60 (m, 4H, C*H*_2_CH_3_), 3.54–3.46 (m, 4H, C*H*_2_CH_3_), 1.24 (t, 12H, ^3^*J*_HH_ = 7.1 Hz, CH_2_C*H*_3_), 0.61 (t, 12H, ^3^*J*_HH_ = 7.0 Hz, CH_2_C*H*_3_) ppm; ^13^C{^1^H}-NMR (101 MHz, C_6_D_6_): *δ* = 131.6 (t, 2C, *J* = 4.4 Hz, C_Ar_), 130.9 (t, 2C, *J* = 8.4 Hz, C_Ar_), 129.9 (t, 2C, *J* = 2.4 Hz, C_Ar_), 129.8 (t, 2C, *J* = 3.3 Hz, C_Ar_), 129.6–129.5 (m, 2C, C_Ar_), 128.3 (s, 2C, C_Ar_), 127.9 (s, 2C, C_Ar_), 127.6 (s, 2C, C_Ar_), 127.5 (s, 2C, C_Ar_), 127.0 (s, 2C, C_Ar_), 125.4–125.1 (m, 4C, C_Ar_), 124.3–124.2 (m, 2C, C_Ar_), 123.6 (t, 2C, *J* = 6.3 Hz, C_Ar_), 63.0–62.8 (m, 4C, *C*H_2_CH_3_), 62.7–62.5 (m, 4C, *C*H_2_CH_3_), 40.5 (t, 2C, ^1^*J*_PC_ = 134 Hz, CH (partially covered by DMSO-d_6_ signal)), 16.3–16.1 (m, 4C, CH_2_*C*H_3_), 15.7–15.5 (m, 4C, CH_2_*C*H_3_) ppm; ^31^P{^1^H}-NMR (202 MHz, C_6_D_6_): *δ* = 18.9 (s, 4P) ppm; IR (ATR) $\tilde{\nu }$ = 1221 (P=O), 1100 (P–OEt) cm^−1^; Anal. Calcd for C_46_H_58_Br_2_N_3_LaO_21_P_4_: C, 39.14; H, 4.14; N 2.98. Found: C, 39.61; H, 4.24; N, 3.19.

### 4.2. Crystallographic Details

X-ray diffraction experiments were performed with either a STOE IPDS 2 with an image plate (Ø 34 cm) using a Mo-GENIX source (λ = 0.71073 nm) or a STOE StadiVari instrument with DECTRIS PILATUS 200 K using a Cu-GENIX source (λ = 1.54186 nm). All structures were solved using direct methods (SHELXT) [60] and refined against F^2^ using the full-matrix least-squares methods of SHELXL [61] within the SHELXLE GUI [62] or with OLEX2 [63]. CCDC 2201668 (**5**) and 2201669 (**6**) contain the supplementary crystallographic data for this paper. These data can be obtained free of charge via www.ccdc.cam.ac.uk/data_request/cif.

### 4.3. Micro-Photoluminescence (µ-PL) Measurments

Luminescence characteristics of phosphonate ester-supported Nd^3+^ complexes are investigated by µ-PL spectroscopy. For the characterization of Nd^3+^ complexes in solid form, the samples were mounted in a liquid helium flow cryostat. The compounds are attached to silicon wafer pieces by partially melting or, if non-meltable, by sticking with vacuum grease, to fix the solid in position and to ensure good thermal conductivity when cooling the sample down to liquid helium temperature. For RT PL measurements in solution, the complexes were dissolved in dry DCM, filled into a cuvette, and attached to the holder of an open cryostat. The Nd^3+^ complexes are excited at 750 nm, using a CW Ti:Sapphire laser. A microscope objective (NA = 0.7) focuses the laser onto the sample and collects the photoluminescence light of the complexes. The emitted light is guided to a monochromator equipped with a liquid nitrogen-cooled InGaAs detector. For polarization-dependent measurements, a polarizer is inserted in front of the detector.

**Figure 10:**
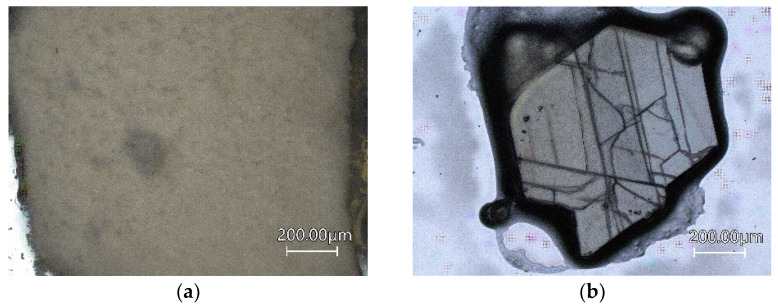
The light microscope images of samples of [Nd(**L3**)_2_(NO_3_)_3_] (5): (**a**) amorphous bulk material; (**b**) crystalline sample.

Low-temperature measurements were conducted on amorphous solids except for [Nd(**L3**)_2_(NO_3_)_3_] (**5**) where a crystalline sample was used. From this compound, crystals were obtained from vapor diffusion of pentanes into a saturated THF solution of the complex. The amorphous bulk material as well as a crystalline sample are depicted in Figure 10a,b.

## 5. Conclusions

The preparation of two geminal bisphosphonate ester-supported Ln^3+^ complexes [Ln(**L3**)_2_(NO_3_)_3_] (Ln = Nd^3+^, La^3+^) has been presented. Emission intensities of equimolar solutions of the germinal bisphosphonate ester-supported Nd^3+^ nitrate complex **5** and related NO_3_^−^ based Nd^3+^ complexes featuring mono-phosphonate esters were compared obtaining unexpected higher emission intensities for the latter compounds. Emission bands from ^4^F_5/2_, ^2^H_9/2_ → ^4^I_J_ (J = 11/2, 13/2) transitions were detected, which are rarely presented for Nd^3+^ containing materials. The three emission bands characteristic for transitions from ^4^F_3/2_ excited state, of mono- and dimeric phosphonate ester-supported Nd^3+^ nitrate and chloride complexes as well as of the geminal bisphosphonate-based complex at liquid helium temperature (5 K) were examined. PL spectra of all three complex types depict similar features with slight shifts of peak positions. Temperature-dependent PL spectroscopy enabled assignment of the transition lines corresponding to the ^4^F_3/2_ → ^4^I_9/2_ transition. At 5 K polarization-dependence of a crystalline sample was observed showing opposite change in peak intensity of transitions related to the depopulation of the R_1_ and R_2_ Stark sublevel, respectively.

This study shows that the investigated neodymium(III) complexes exhibit interesting luminescence properties. With improved synthesis processes, their optical properties could be further enhanced. In the next step, molecules will be integrated onto microcavities to examine molecule-cavity coupling.

## Figures and Tables

**Table 1:** A summary of λ_max_ values of **1**–**5** of transition lines corresponding to the ^4^F_3/2_ → ^4^I_9/2_ transition.

Complex	[Nd(L1)_3_(NO_3_)_3_] (1)	[Nd(L1)_3_Cl_3_]_2_ (2)	[Nd(L2)_3_(NO_3_)_3_] (3)	[Nd(L2)_3_Cl_3_]_2_ (4)	[Nd(L3)_2_(NO_3_)_3_] (5)
Transition	*λ_max_* [nm]				
R_2_ → Z_1_	867.87	873.55	869.29	875.45	863.92
R_1_ → Z_1_	871.26	879.41	872.29	878.38	-
R_2_ → Z_2_	873.28	-	875.06	-	868.21
R_1_ → Z_2_	876.54	-	878.06	881.49	871.95
R_2_ → Z_3_	879.29	-	879.66	883.36	876.61
R_1_ → Z_3_	882.74	-	882.73	886.56	880.89
R_2_ → Z_4_	891.55	-	892.53	891.67	892.21
R_1_ → Z_4_	895.34	-	895.60	894.46	-
R_2_ → Z_5_	901.99	900.21	901.19	901.48	897.14
R_1_ → Z_5_	905.56	906.46	904.19	904.68	901.97

## Data Availability

NMR, IR, and crystallographic data presented in this study are available in the Supplementary Materials.

## Supplementary Materials

The following are available online at https://www.mdpi.com/article/10.3390/molecules28010048/s1, Figure S1: ^1^H NMR spectrum of **6** in DMSO-d_6_, Figure S2: ^13^C{^1^H} NMR spectrum of **6** in DMSO-d_6_, Table S1: Crystallographic data for complex **5** and **6**, Figure S3: Asymmetric unit of **5**, Figure S4: Asymmetric unit of **6**, Figure S5: ATR IR-spectrum of complex [Nd(**L3**)_2_(NO_3_)_3_] (**5**), Figure S6: ATR IR-spectrum of complex [La(**L3**)_2_(NO_3_)_3_] (**6**), Figure S7: Normalized absorption spectrum of complex [Nd(**L3**)_2_(NO_3_)_3_] (**5**) at room temperature showing sharp Nd^3+^ absorption bands. Reference [64] is cited in the Supplementary Materials.

## Sample Availability

Samples of the compounds are not available from the authors.
