# Supplementary material for: Luminescent Properties of Phosphonate Ester-Supported Neodymium(III) Nitrate and Chloride Complexes
Source: Molecules. 2022 Dec 21;28(1):48. doi: 10.3390/molecules28010048 (PMC9822372; doi:10.3390/molecules28010048)
Supplement: Supplementary file 1 [file molecules-28-00048-s001.zip › molecules-2092638-supplementary.pdf]

Supporting Information

Luminescent properties of phosphonate ester-supported neodymium(III) nitrate and chloride complexes

Miriam Gerstel [1], Ingo Koehne [2], Johann Peter Reithmaier[1], Rudolf Pietschnig [2], and Mohamed Benyoucef [1*]

[1]: Institute of Nanostructure Technologies and Analytics (INA) and CINSaT, University of Kassel, Heinrich-Plett-Str. 40, 34132 Kassel, Germany.

[2]: Institute of Chemistry and Center for Interdisciplinary Nanostructure Science and Technology (CINSaT), University of Kassel, Heinrich-Plett-Str. 40, 34132 Kassel, Germany.

[*]Email: m.benyoucef@physik.uni-kassel.de

Contents

1 NMR Spectra ... 2

1.1 Geminal Bisphosphonate Ester Complex $[La(L3)_2(NO_3)_3]$ (6) ... 2

2 Crystallographic Data ... 3

2.1 Complex $[Nd(L3)_2(NO_3)_3]$ (5) ... 4

2.2 Complex $[La(L3)_2(NO_3)_3]$ (6) ... 8

3 ATR IR Spectra of Complexes 5 & 6 ... 12

4 Absorption Spectrum of Complex 5 ... 13

5 References ... 13

1 NMR Spectra

1.1 Geminal Bisphosphonate Ester Complex [La(L3)$_2$(NO$_3$)$_3$] (6)

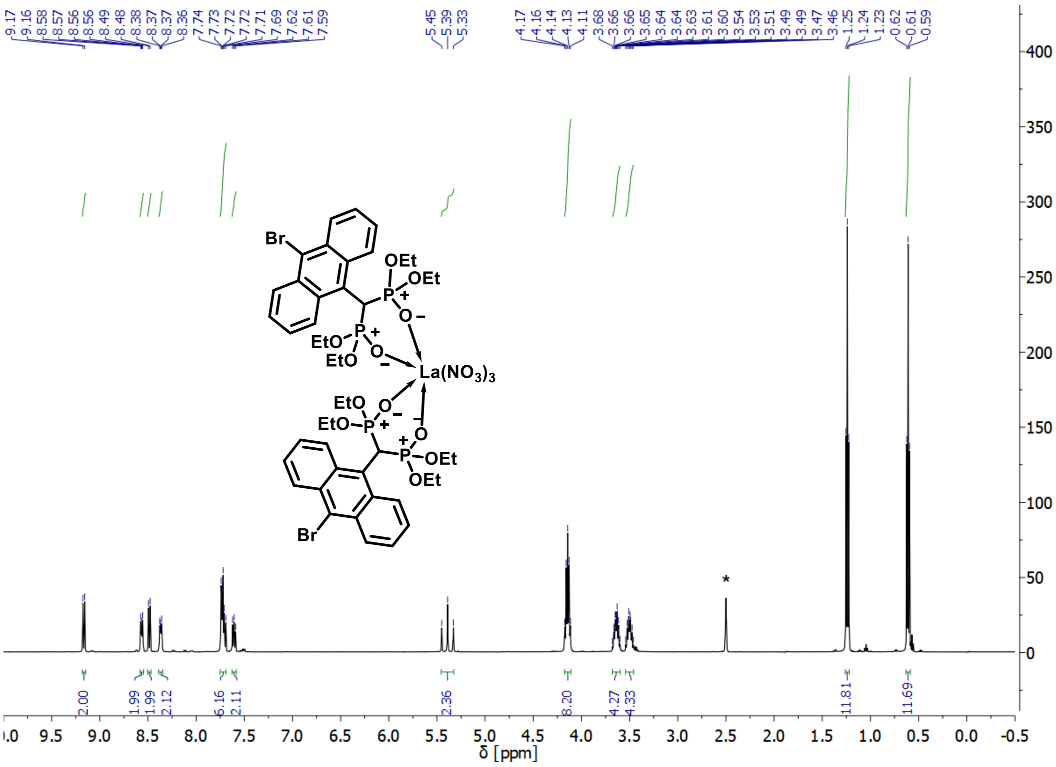

Figure S1. *^{1}H-NMR spectrum of **6** in DMSO-d$_6$ (*).*

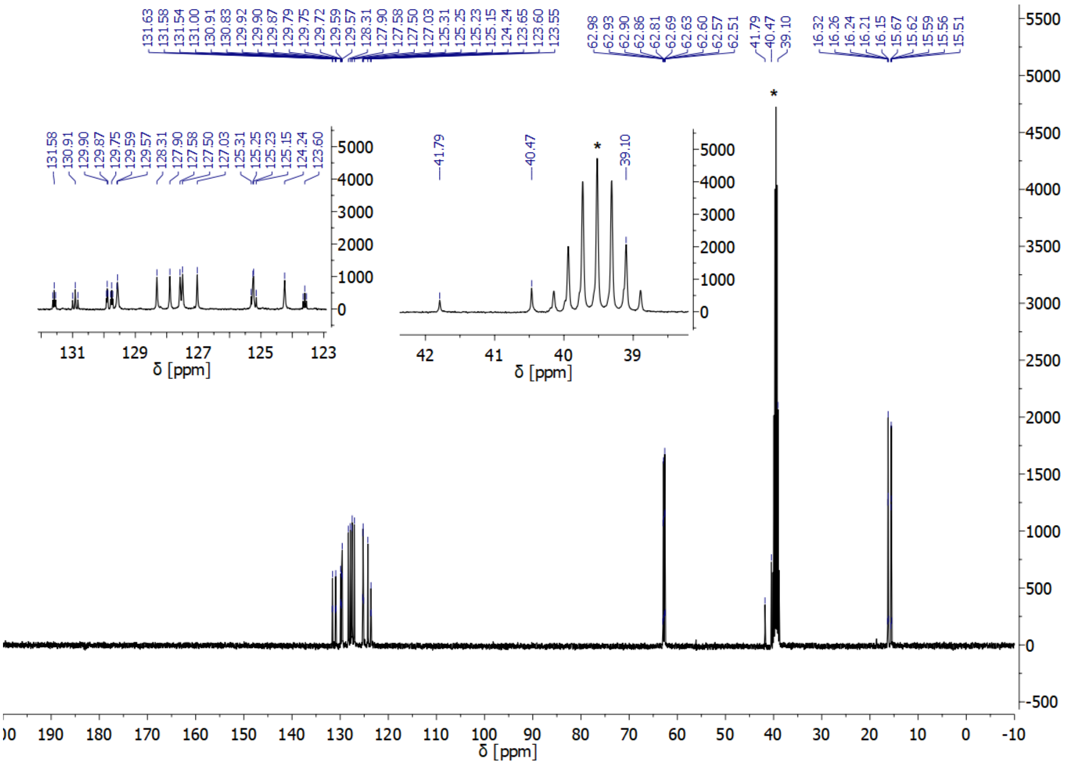

Figure S2. *^{13}C{^{1}H}-NMR spectrum of **6** in DMSO-d$_6$ (*).*

2 CRYSTALLOGRAPHIC DATA

Table S1. *Crystallographic data for complexes **5** and **6**.*

**Compound**	**5**	**6**
CCDC number	2201668	2201669
formula	$C_{46}H_{58}Br_2N_3NdO_{21}P_4$	$C_{46}H_{58}Br_2LaN_3O_{21}P_4$
MW [g mol^{-1}]	1416.89	1411.56
T [K]	100(2)	100(2)
space group	C2/*c*	*C*2/*c*
a [Å]	33.0143(18)	33.096(2)
b [Å]	8.7779(3)	8.8402(4)
c [Å]	19.2500(12)	19.2891(12)
α [°]	90	90
β [°]	99.176(5)	98.866(5)
γ [°]	90	90
V [Å]^3	5507.2(5)	5576.0(6)
Z	4	4
μ [mm^{-1}]	2.589	9.361
θ_{max} [°]	25.776	71.133
reflections measured	12710	10096
reflections unique	5232	5049
R_{int}	0.049	0.036
data/restr./para.	5232/140/522	3808/0/229
R_1 [I>2σ(I)][a]	0.0640	0.0629
wR_2 (all)[b]	0.1833	0.1614
Abs. struct. param.[1]	0.00082(15)	--
$\Delta\rho$fin [eÅ^{-3}]	1.04/–1.45	1.04/–1.27

[a] $R_1 = (\Sigma|F_o|-|F_c|)/(\Sigma|F_o|)$; [b] $wR_2 = [\{\Sigma w(F_o^2-F_c^2)2\}/\{\Sigma w(F_o^2)^2\}]^{1/2}$.

2.1 Complex $[Nd(L3)_2(NO_3)_3]$ (5)

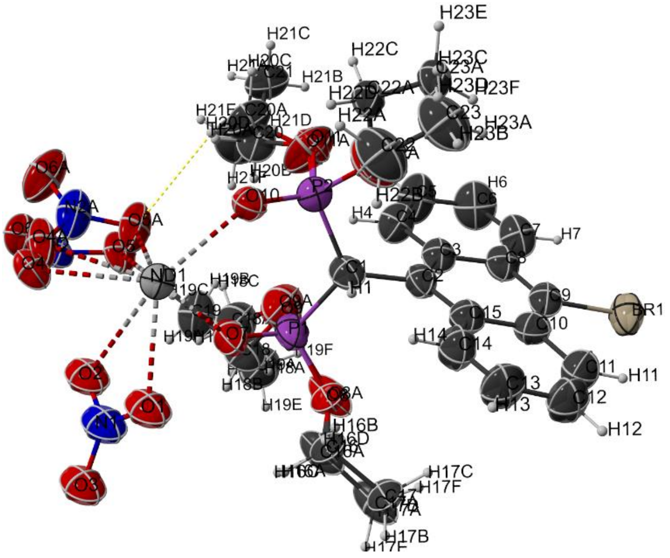

Figure S3. *Asymmetric unit of **5**.*

5 crystallizes in the monoclinic space group *C*2/*c* containing half a molecule in the asymmetric unit. The other half is generated by a two-fold rotational axis (Symmetry code: #1: -x+1 ,y, -z+3/2). The Nd ion as well as one of the nitrate anions reside on a special position on a C_2 symmetry element and hence are only occupied by ½. The latter also shows a special position disorder about two positions. The other nitrate anion and the four ethoxy groups are disordered about two positions as well. They are refined with distance restraints and restraints for the anisotropic displacement parameters. The minor positions refined to 0.499(15), 0.436(18) and 0.497(12), respectively.

Bond lengths [Å] and angles [°] of **5**.

O(1)–N(1)	1.262(13)	Nd(1)–O(5)#1	2.522(10)
O(1)–Nd(1)	2.479(11)	Nd(1)–O(5A)#1	2.606(11)
O(2)–N(1)	1.272(13)	Nd(1)–O(5A)	2.607(11)
O(2)–Nd(1)	2.570(10)	Nd(1)–O(4)#1	2.627(11)
N(1)–O(3)	1.241(12)	Nd(1)–O(4)	2.627(11)
Br(1)–C(9)	1.896(7)	O(4)–N(2)	1.270(15)
C(1)–C(2)	1.580(9)	O(5)–N(2)	1.277(13)
C(1)–P(2)	1.772(7)	N(2)–O(6)	1.219(11)
C(1)–P(1)	1.786(6)	O(4A)–N(2A)	1.258(15)
Nd(1)–O(10)	2.412(4)	O(5A)–N(2A)	1.266(13)
Nd(1)–O(10)#1	2.413(4)	N(2A)–O(6A)	1.276(13)
Nd(1)–O(7)#1	2.473(5)	P(1)–O(7)	1.468(5)
Nd(1)–O(7)	2.473(5)	P(1)–O(8)	1.543(8)
Nd(1)–O(5)	2.522(10)	P(1)–O(9)	1.551(9)

P(1)–O(8A)	1.555(14)		
P(1)–O(9A)	1.560(15)	N(1)–O(1)–Nd(1)	98.5(8)
O(8)–C(16)	1.432(14)	N(1)–O(2)–Nd(1)	93.9(8)
C(16)–C(17)	1.541(13)	O(3)–N(1)–O(1)	120.4(11)
O(9)–C(18)	1.456(14)	O(3)–N(1)–O(2)	122.6(12)
C(18)–C(19)	1.448(19)	O(1)–N(1)–O(2)	116.9(10)
O(8A)–C(16A)	1.438(16)	O(3)–N(1)–Nd(1)	176.8(10)
C(16A)–C(17A)	1.523(17)	O(1)–N(1)–Nd(1)	56.4(6)
O(9A)–C(18A)	1.446(18)	O(2)–N(1)–Nd(1)	60.6(7)
C(18A)–C(19A)	1.47(2)	C(2)–C(1)–P(2)	121.7(4)
P(2)–O(10)	1.463(5)	C(2)–C(1)–P(1)	115.9(4)
P(2)–O(11A)	1.549(12)	P(2)–C(1)–P(1)	118.4(4)
P(2)–O(11)	1.558(9)	O(10)–Nd(1)–O(10)#1	71.2(2)
P(2)–O(12)	1.564(13)	O(10)–Nd(1)–O(7)#1	136.05(17)
P(2)–O(12A)	1.564(8)	O(10)#1–Nd(1)–O(7)#1	72.51(16)
O(11)–C(20)	1.467(15)	O(10)–Nd(1)–O(7)	72.51(16)
C(20)–C(21)	1.502(19)	O(10)#1–Nd(1)–O(7)	136.05(17)
O(12)–C(22)	1.405(19)	O(7)#1–Nd(1)–O(7)	150.1(2)
C(22)–C(23)	1.383(17)	O(10)–Nd(1)–O(1)	135.9(3)
O(11A)–C(20A)	1.467(16)	O(10)#1–Nd(1)–O(1)	147.2(3)
C(20A)–C(21A)	1.494(19)	O(7)#1–Nd(1)–O(1)	74.7(3)
O(12A)–C(22A)	1.432(16)	O(7)–Nd(1)–O(1)	76.6(3)
C(22A)–C(23A)	1.426(14)	O(10)–Nd(1)–O(5)	82.7(3)
C(2)–C(3)	1.387(10)	O(10)#1–Nd(1)–O(5)	80.6(3)
C(2)–C(15)	1.405(9)	O(7)#1–Nd(1)–O(5)	114.8(3)
C(3)–C(4)	1.430(10)	O(7)–Nd(1)–O(5)	70.9(3)
C(3)–C(8)	1.450(9)	O(1)–Nd(1)–O(5)	115.8(4)
C(4)–C(5)	1.359(12)	O(10)–Nd(1)–O(5)#1	80.6(3)
C(5)–C(6)	1.392(13)	O(10)#1–Nd(1)–O(5)#1	82.7(3)
C(6)–C(7)	1.359(12)	O(7)#1–Nd(1)–O(5)#1	70.9(3)
C(7)–C(8)	1.420(11)	O(7)–Nd(1)–O(5)#1	114.8(3)
C(8)–C(9)	1.374(10)	O(1)–Nd(1)–O(5)#1	84.8(4)
C(9)–C(10)	1.388(11)	O(5)–Nd(1)–O(5)#1	159.3(6)
C(10)–C(15)	1.437(10)	O(10)–Nd(1)–O(2)	139.7(3)
C(10)–C(11)	1.444(10)	O(10)#1–Nd(1)–O(2)	125.7(3)
C(11)–C(12)	1.340(12)	O(7)#1–Nd(1)–O(2)	82.6(3)
C(12)–C(13)	1.397(13)	O(7)–Nd(1)–O(2)	72.6(3)
C(13)–C(14)	1.271(10)	O(1)–Nd(1)–O(2)	50.6(4)
C(14)–C(15)	1.505(10)	O(5)–Nd(1)–O(2)	67.2(4)

O(5)#1–Nd(1)–O(2)	133.1(4)	N(2A)–O(4A)–Nd(1)	95.6(7)
O(10)–Nd(1)–O(5A)#1	72.1(3)	N(2A)–O(5A)–Nd(1)	100.2(9)
O(10)#1–Nd(1)–O(5A)#1	70.3(4)	O(4A)–N(2A)–O(5A)	114.6(12)
O(7)#1–Nd(1)–O(5A)#1	72.9(3)	O(4A)–N(2A)–O(6A)	121.6(12)
O(7)–Nd(1)–O(5A)#1	119.9(3)	O(5A)–N(2A)–O(6A)	123.8(15)
O(1)–Nd(1)–O(5A)#1	98.1(5)	O(7)–P(1)–O(8)	111.5(7)
O(5)–Nd(1)–O(5A)#1	146.1(6)	O(7)–P(1)–O(9)	116.1(8)
O(5)#1–Nd(1)–O(5A)#1	13.4(3)	O(8)–P(1)–O(9)	104.0(10)
O(2)–Nd(1)–O(5A)#1	145.0(4)	O(7)–P(1)–O(8A)	117.1(9)
O(10)–Nd(1)–O(5A)	70.3(4)	O(7)–P(1)–O(9A)	114.3(14)
O(10)#1–Nd(1)–O(5A)	72.1(3)	O(8A)–P(1)–O(9A)	102.5(13)
O(7)#1–Nd(1)–O(5A)	119.9(3)	O(7)–P(1)–C(1)	111.2(3)
O(7)–Nd(1)–O(5A)	72.9(3)	O(8)–P(1)–C(1)	107.7(10)
O(5A)#1–Nd(1)–O(5A)	133.3(8)	O(9)–P(1)–C(1)	105.8(6)
O(10)–Nd(1)–O(4)#1	71.5(3)	O(8A)–P(1)–C(1)	108.3(13)
O(10)#1–Nd(1)–O(4)#1	123.8(3)	O(9A)–P(1)–C(1)	102.2(9)
O(7)#1–Nd(1)–O(4)#1	111.0(4)	C(16)–O(8)–P(1)	122.8(11)
O(7)–Nd(1)–O(4)#1	64.3(3)	O(8)–C(16)–C(17)	106.7(11)
O(1)–Nd(1)–O(4)#1	67.1(4)	C(18)–O(9)–P(1)	114.9(11)
O(5)–Nd(1)–O(4)#1	133.0(4)	C(19)–C(18)–O(9)	106.4(15)
O(5)#1–Nd(1)–O(4)#1	51.0(3)	C(16A)–O(8A)–P(1)	119.3(14)
O(2)–Nd(1)–O(4)#1	109.9(4)	O(8A)–C(16A)–C(17A)	108.6(14)
O(10)–Nd(1)–O(4)	123.8(3)	C(18A)–O(9A)–P(1)	128(2)
O(10)#1–Nd(1)–O(4)	71.5(3)	O(9A)–C(18A)–C(19A)	112(2)
O(7)#1–Nd(1)–O(4)	64.3(3)	O(10)–P(2)–O(11A)	113.3(9)
O(7)–Nd(1)–O(4)	111.0(4)	O(10)–P(2)–O(11)	115.7(7)
O(1)–Nd(1)–O(4)	96.2(4)	O(10)–P(2)–O(12)	117.6(9)
O(5)–Nd(1)–O(4)	51.0(3)	O(11)–P(2)–O(12)	98.5(11)
O(5)#1–Nd(1)–O(4)	133.0(4)	O(10)–P(2)–O(12A)	114.5(6)
O(2)–Nd(1)–O(4)	54.3(4)	O(11A)–P(2)–O(12A)	106.0(10)
O(4)#1–Nd(1)–O(4)	163.1(6)	O(10)–P(2)–C(1)	109.2(3)
N(2)–O(4)–Nd(1)	91.5(7)	O(11A)–P(2)–C(1)	110.7(12)
N(2)–O(5)–Nd(1)	96.2(7)	O(11)–P(2)–C(1)	112.7(9)
O(6)–N(2)–O(4)	120.6(11)	O(12)–P(2)–C(1)	102.1(12)
O(6)–N(2)–O(5)	118.4(12)	O(12A)–P(2)–C(1)	102.6(7)
O(4)–N(2)–O(5)	121.0(10)	C(20)–O(11)–P(2)	120.3(11)
O(6)–N(2)–Nd(1)	173.8(10)	O(11)–C(20)–C(21)	103.8(14)
O(4)–N(2)–Nd(1)	63.0(6)	C(22)–O(12)–P(2)	125.2(18)
O(5)–N(2)–Nd(1)	58.2(6)	C(23)–C(22)–O(12)	117.1(16)

C(20A)–O(11A)–P(2)	123.3(16)	C(9)–C(8)–C(3)	117.7(7)
O(11A)–C(20A)–C(21A)	108.5(17)	C(7)–C(8)–C(3)	118.9(6)
C(22A)–O(12A)–P(2)	124.8(11)	C(8)–C(9)–C(10)	123.5(6)
C(23A)–C(22A)–O(12A)	112.2(10)	C(8)–C(9)–Br(1)	118.3(6)
C(3)–C(2)–C(15)	121.1(6)	C(10)–C(9)–Br(1)	118.1(5)
C(3)–C(2)–C(1)	119.0(6)	P(2)–O(10)–Nd(1)	148.8(3)
C(15)–C(2)–C(1)	119.9(6)	C(9)–C(10)–C(15)	118.5(6)
C(2)–C(3)–C(4)	123.1(6)	C(9)–C(10)–C(11)	123.5(7)
C(2)–C(3)–C(8)	119.9(6)	C(15)–C(10)–C(11)	117.9(7)
C(4)–C(3)–C(8)	116.9(6)	C(12)–C(11)–C(10)	123.1(8)
C(5)–C(4)–C(3)	121.3(7)	C(11)–C(12)–C(13)	119.0(8)
C(4)–C(5)–C(6)	121.1(8)	C(14)–C(13)–C(12)	122.2(9)
C(7)–C(6)–C(5)	120.6(8)	C(13)–C(14)–C(15)	123.7(8)
P(1)–O(7)–Nd(1)	143.8(3)	C(2)–C(15)–C(10)	119.0(6)
C(6)–C(7)–C(8)	121.0(8)	C(2)–C(15)–C(14)	127.1(6)
C(9)–C(8)–C(7)	123.4(7)	C(10)–C(15)–C(14)	113.8(6)

2.2 Complex $[La(L3)_2(NO_3)_3]$ (6)

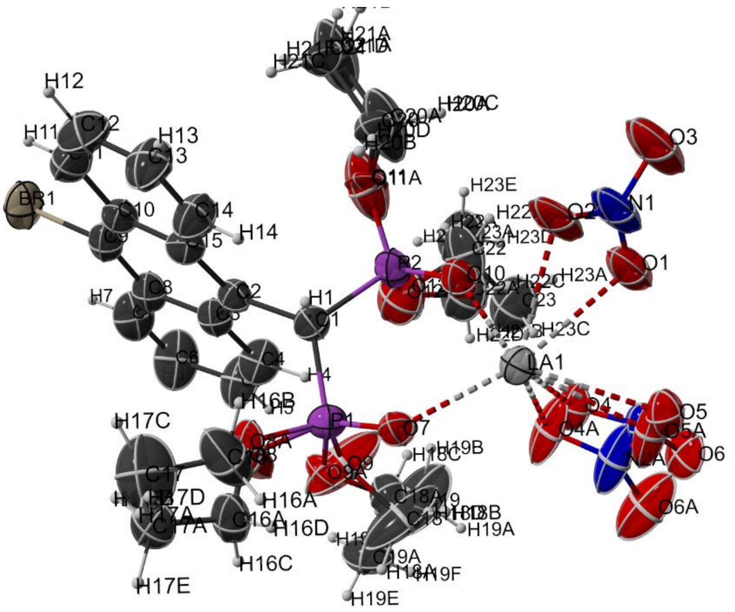

Figure S4. *Asymmetric unit of **6**.*

6 crystallizes in the monoclinic space group *C*2/*c* containing half a molecule in the asymmetric unit. The other half is generated by a two-fold rotational axis (Symmetry code: #1: -x+1 ,y, -z+3/2). The La ion as well as one of the nitrate anions reside on a special position on a C_2 symmetry element and hence are only occupied by ½. The latter also shows a special position disorder about two positions. The other nitrate anion and the four ethoxy groups are disordered about two positions as well. They are refined with distance restraints and restraints for the anisotropic displacement parameters. The minor positions refined to 0.477(22), 0.471(15) and 0.363(15), respectively.

Bond lengths [Å] and angles [°] of **6**.

C(1)–C(2)	1.555(9)	La(1)–O(7)	2.466(5)
C(1)–P(1)	1.780(7)	La(1)–O(7)#1	2.466(5)
C(1)–P(2)	1.796(7)	La(1)–O(10)	2.538(5)
O(1)–N(1)	1.283(15)	La(1)–O(10)#1	2.538(5)
O(1)–La(1)	2.583(17)	La(1)–O(4)	2.609(10)
O(2)–N(1)	1.284(17)	La(1)–O(4)#1	2.609(10)
O(2)–La(1)	2.582(18)	La(1)–O(4A)	2.658(13)
N(1)–O(3)	1.217(13)	La(1)–O(4A)#1	2.658(13)
C(3)–C(2)	1.418(10)	La(1)–O(5)	2.667(12)
C(3)–C(4)	1.426(10)	La(1)–O(5)#1	2.667(12)
C(3)–C(8)	1.433(9)	O(4)–N(2)	1.273(13)
C(2)–C(15)	1.412(9)	O(5)–N(2)	1.274(15)

N(2)–O(6)	1.211(12)	C(11)–C(12)	1.341(13)
O(4A)–N(2A)	1.263(14)	C(12)–C(13)	1.402(14)
O(5A)–N(2A)	1.276(18)	C(13)–C(14)	1.269(11)
N(2A)–O(6A)	1.244(15)	C(14)–C(15)	1.510(10)
C(4)–C(5)	1.350(11)		
C(5)–C(6)	1.414(13)	C(2)–C(1)–P(1)	121.4(5)
P(1)–O(7)	1.466(5)	C(2)–C(1)–P(2)	116.0(5)
P(1)–O(8)	1.539(13)	P(1)–C(1)–P(2)	117.8(4)
P(1)–O(9A)	1.540(9)	N(1)–O(1)–La(1)	96.1(11)
P(1)–O(9)	1.541(13)	N(1)–O(2)–La(1)	96.1(11)
P(1)–O(8A)	1.567(8)	O(3)–N(1)–O(1)	122.4(16)
O(8)–C(16)	1.49(2)	O(3)–N(1)–O(2)	120.1(14)
C(16)–C(17)	1.34(2)	O(1)–N(1)–O(2)	117.4(11)
O(9)–C(18)	1.450(18)	O(3)–N(1)–La(1)	178.8(15)
C(18)–C(19)	1.47(2)	O(1)–N(1)–La(1)	58.8(9)
O(8A)–C(16A)	1.463(18)	O(2)–N(1)–La(1)	58.7(9)
C(16A)–C(17A)	1.375(17)	C(2)–C(3)–C(4)	122.6(6)
O(9A)–C(18A)	1.443(14)	C(2)–C(3)–C(8)	120.2(6)
C(18A)–C(19A)	1.44(2)	C(4)–C(3)–C(8)	117.1(6)
C(6)–C(7)	1.352(13)	C(15)–C(2)–C(3)	119.3(6)
C(7)–C(8)	1.440(11)	C(15)–C(2)–C(1)	120.8(6)
P(2)–O(10)	1.467(5)	C(3)–C(2)–C(1)	119.9(6)
P(2)–O(11A)	1.551(17)	O(7)–La(1)–O(7)#1	71.7(2)
P(2)–O(12)	1.554(8)	O(7)–La(1)–O(10)	71.43(16)
P(2)–O(12A)	1.559(16)	O(7)#1–La(1)–O(10)	135.24(17)
P(2)–O(11)	1.561(7)	O(7)–La(1)–O(10)#1	135.24(17)
O(11)–C(20)	1.410(14)	O(7)#1–La(1)–O(10)#1	71.43(15)
C(20)–C(21)	1.549(14)	O(10)–La(1)–O(10)#1	152.0(2)
O(12)–C(22)	1.462(14)	O(7)–La(1)–O(2)	135.3(4)
C(22)–C(23)	1.40(2)	O(7)#1–La(1)–O(2)	146.3(4)
O(11A)–C(20A)	1.404(19)	O(10)–La(1)–O(2)	78.4(4)
C(20A)–C(21A)	1.55(2)	O(10)#1–La(1)–O(2)	75.0(4)
O(12A)–C(22A)	1.44(2)	O(7)–La(1)–O(1)	140.3(4)
C(22A)–C(23A)	1.42(3)	O(7)#1–La(1)–O(1)	126.8(4)
C(8)–C(9)	1.391(11)	O(10)–La(1)–O(1)	73.2(4)
C(9)–C(10)	1.389(11)	O(10)#1–La(1)–O(1)	83.2(4)
C(9)–Br(1)	1.903(7)	O(2)–La(1)–O(1)	50.3(4)
C(10)–C(15)	1.425(9)	O(7)–La(1)–O(4)	83.2(3)
C(10)–C(11)	1.429(11)	O(7)#1–La(1)–O(4)	79.2(3)

O(10)–La(1)–O(4)	72.1(3)	O(5)–La(1)–O(5)#1	165.6(7)
O(10)#1–La(1)–O(4)	113.5(3)	N(2)–O(4)–La(1)	97.6(7)
O(2)–La(1)–O(4)	118.0(4)	N(2)–O(5)–La(1)	94.8(7)
O(1)–La(1)–O(4)	69.2(4)	O(6)–N(2)–O(4)	119.7(12)
O(7)–La(1)–O(4)#1	79.2(3)	O(6)–N(2)–O(5)	122.1(12)
O(7)#1–La(1)–O(4)#1	83.2(3)	O(4)–N(2)–O(5)	118.3(10)
O(10)–La(1)–O(4)#1	113.5(3)	O(6)–N(2)–La(1)	173.8(11)
O(10)#1–La(1)–O(4)#1	72.1(3)	O(4)–N(2)–La(1)	58.0(6)
O(2)–La(1)–O(4)#1	83.7(4)	O(5)–N(2)–La(1)	60.6(6)
O(1)–La(1)–O(4)#1	132.3(5)	N(2A)–O(4A)–La(1)	99.0(9)
O(4)–La(1)–O(4)#1	158.3(6)	N(2A)–O(5A)–La(1)	96.5(8)
O(7)–La(1)–O(4A)	71.0(5)	O(6A)–N(2A)–O(4A)	124.2(18)
O(7)#1–La(1)–O(4A)	71.4(5)	O(6A)–N(2A)–O(5A)	121.2(15)
O(10)–La(1)–O(4A)	73.2(4)	O(4A)–N(2A)–O(5A)	114.4(14)
O(10)#1–La(1)–O(4A)	118.7(3)	C(5)–C(4)–C(3)	122.6(8)
O(7)–La(1)–O(4A)#1	71.4(5)	C(4)–C(5)–C(6)	120.1(8)
O(7)#1–La(1)–O(4A)#1	71.0(5)	O(7)–P(1)–O(8)	119.5(9)
O(10)–La(1)–O(4A)#1	118.7(3)	O(7)–P(1)–O(9A)	117.4(6)
O(10)#1–La(1)–O(4A)#1	73.2(4)	O(7)–P(1)–O(9)	108.3(9)
O(2)–La(1)–O(4A)#1	96.5(7)	O(8)–P(1)–O(9)	104.2(13)
O(1)–La(1)–O(4A)#1	143.9(7)	O(7)–P(1)–O(8A)	113.1(6)
O(4)–La(1)–O(4A)#1	145.5(7)	O(9A)–P(1)–O(8A)	102.6(7)
O(4)#1–La(1)–O(4A)#1	13.1(5)	O(7)–P(1)–C(1)	109.0(3)
O(7)–La(1)–O(5)	122.8(3)	O(8)–P(1)–C(1)	106.8(12)
O(7)#1–La(1)–O(5)	70.3(4)	O(9A)–P(1)–C(1)	112.9(6)
O(10)–La(1)–O(5)	111.1(4)	O(9)–P(1)–C(1)	108.5(11)
O(10)#1–La(1)–O(5)	65.1(4)	O(8A)–P(1)–C(1)	100.5(7)
O(2)–La(1)–O(5)	98.4(5)	C(16)–O(8)–P(1)	118.9(16)
O(1)–La(1)–O(5)	56.6(5)	C(17)–C(16)–O(8)	115(2)
O(4)–La(1)–O(5)	48.9(3)	C(18)–O(9)–P(1)	127.8(17)
O(4)#1–La(1)–O(5)	134.7(4)	O(9)–C(18)–C(19)	103.9(19)
O(7)–La(1)–O(5)#1	70.3(4)	C(16A)–O(8A)–P(1)	124.6(12)
O(7)#1–La(1)–O(5)#1	122.8(3)	C(17A)–C(16A)–O(8A)	114.3(13)
O(10)–La(1)–O(5)#1	65.1(4)	C(18A)–O(9A)–P(1)	122.9(10)
O(10)#1–La(1)–O(5)#1	111.1(4)	C(19A)–C(18A)–O(9A)	108.6(13)
O(2)–La(1)–O(5)#1	67.4(5)	C(7)–C(6)–C(5)	120.2(8)
O(1)–La(1)–O(5)#1	109.8(5)	P(1)–O(7)–La(1)	149.1(3)
O(4)–La(1)–O(5)#1	134.7(4)	C(6)–C(7)–C(8)	121.3(8)
O(4)#1–La(1)–O(5)#1	48.9(3)	O(10)–P(2)–O(11A)	115.8(13)

O(10)–P(2)–O(12)	114.6(8)	C(9)–C(8)–C(3)	118.1(7)
O(10)–P(2)–O(12A)	116.2(16)	C(9)–C(8)–C(7)	123.4(7)
O(11A)–P(2)–O(12A)	102.0(16)	C(3)–C(8)–C(7)	118.5(7)
O(10)–P(2)–O(11)	113.1(6)	C(10)–C(9)–C(8)	123.3(7)
O(12)–P(2)–O(11)	103.6(8)	C(10)–C(9)–Br(1)	118.9(6)
O(10)–P(2)–C(1)	111.7(3)	C(8)–C(9)–Br(1)	117.8(5)
O(11A)–P(2)–C(1)	108.8(17)	P(2)–O(10)–La(1)	143.6(3)
O(12)–P(2)–C(1)	105.8(5)	C(9)–C(10)–C(15)	118.3(6)
O(12A)–P(2)–C(1)	100.9(12)	C(9)–C(10)–C(11)	122.9(7)
O(11)–P(2)–C(1)	107.4(8)	C(15)–C(10)–C(11)	118.9(7)
C(20)–O(11)–P(2)	121.6(9)	C(12)–C(11)–C(10)	122.9(8)
O(11)–C(20)–C(21)	107.9(10)	C(11)–C(12)–C(13)	119.4(8)
C(22)–O(12)–P(2)	116.3(10)	C(14)–C(13)–C(12)	120.9(9)
C(23)–C(22)–O(12)	109.8(17)	C(13)–C(14)–C(15)	124.7(8)
C(20A)–O(11A)–P(2)	121(2)	C(2)–C(15)–C(10)	120.6(6)
O(11A)–C(20A)–C(21A)	107(2)	C(2)–C(15)–C(14)	126.3(6)
C(22A)–O(12A)–P(2)	127(3)	C(10)–C(15)–C(14)	113.1(6)
C(23A)–C(22A)–O(12A)	113(3)		

3 ATR IR SPECTRA OF COMPLEXES 5 & 6

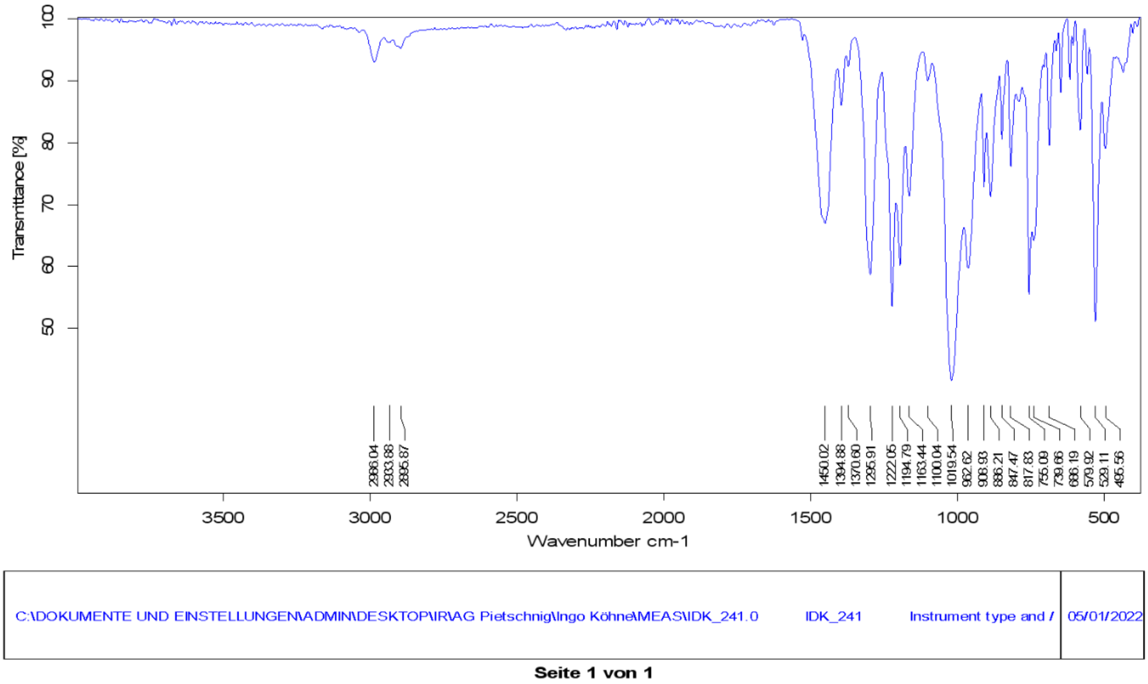

***Figure S5**. ATR IR-spectrum of complex [Nd(**L3**)$_2$(NO$_3$)$_3$] (**5**).*

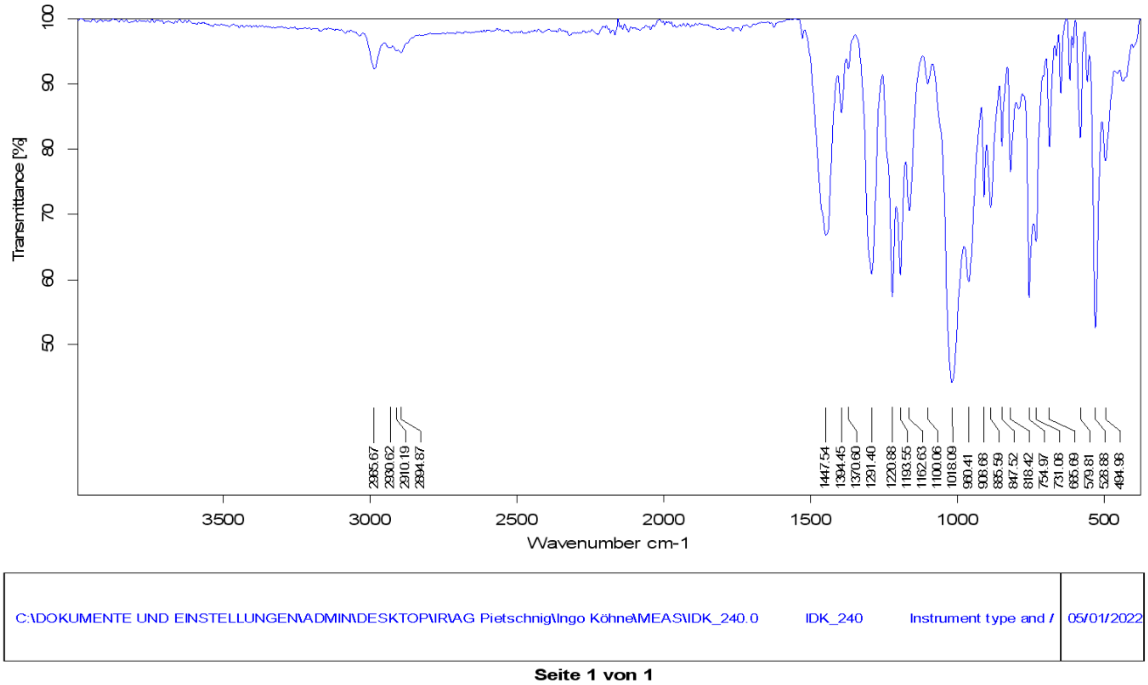

***Figure S6**. ATR IR-spectrum of complex[La(**L3**)$_2$(NO$_3$)$_3$] (**6**).*

4 ABSORPTION SPECTRUM OF COMPLEX 5

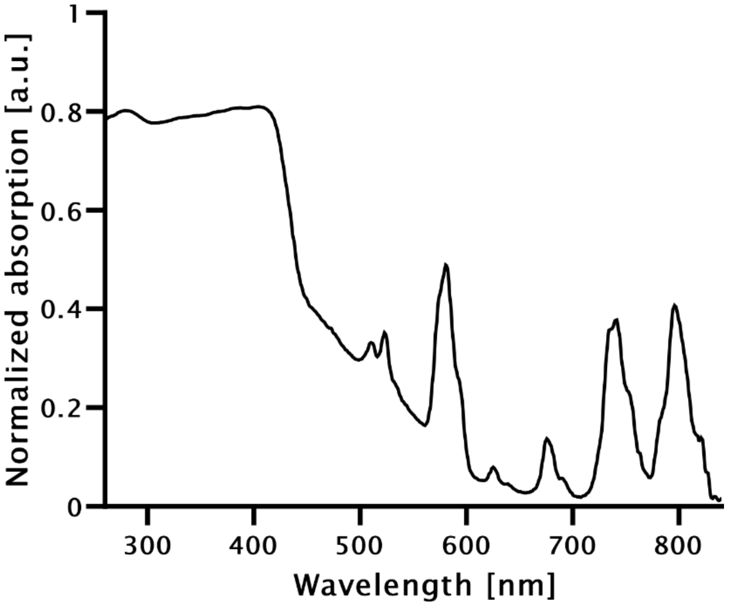

Figure S7. Normalized absorption spectrum of complex *[Nd(**L3**)$_2$(NO$_3$)$_3$] (**5**) at room temperature showing sharp Nd^{3+} absorption bands.*

5 REFERENCES

1. Parsons, S.; Flack, H. D.; Wagner, T., Use of intensity quotients and differences in absolute structure refinement. *Acta Crystallogr. B* **2013,** *69* (3), 249-259.
